# Supplementary material for: Pharmacokinetic Characterization of Supinoxin and Its Physiologically Based Pharmacokinetic Modeling in Rats
Source: Pharmaceutics. 2021 Mar 11;13(3):373. doi: 10.3390/pharmaceutics13030373 (PMC7998731; doi:10.3390/pharmaceutics13030373)
Supplement: Supplementary file 1 [file pharmaceutics-13-00373-s001.pdf]

pharmaceutics

MDPI

Supplementary Materials: Pharmacokinetic Characterization of Supinoxin and its Physiologically Based Pharmacokinetic Modeling in Rats

Yoo-Kyung Song, Yun-Hwan Seol, Min Ju Kim, Jong-Woo Jeong, Hae-In Choi, Seung-Won Lee, Yoon-Jee Chae, Sunjoo Ahn, Young-Dae Gong, Kyeong-Ryoon Lee* and Tae-Sung Koo *

Table S1. Plasma concentrations of supinoxin after intravenous injection of 0.5, 1 and 5 mg/kg in fasted male SD rats.

(A) 0.5 mg/kg.

	Plasma concentration (µg/mL)						
**Time (h)**	**#1**	**#2**	**#3**	**#4**	**#5**	**Mean**	**S.D.**
0.083	0.3220	0.3380	0.3850	0.2300	0.2910	0.3132	0.0576
0.25	0.2580	0.2990	0.3100	0.1780	0.2290	0.2548	0.0538
0.5	0.2320	0.2370	0.2450	0.1260	0.1920	0.2064	0.0494
1	0.1080	0.1400	0.1930	0.0982	0.1590	0.1396	0.0385
3	0.0812	0.0773	0.1140	0.0483	0.0758	0.0793	0.0234
7	0.0204	0.0220	0.0290	0.0200	0.0128	0.0208	0.0058
10	0.0072	0.0074	0.0126	0.0092	0.0049	0.0082	0.0029
24	BQL	BQL	0.0007	0.0008	BQL	0.0003	0.0004

BQL, Below the quantification limit, treated as "0".

(B) 1 mg/kg.

	Plasma concentration (µg/mL)						
**Time (h)**	**#1**	**#2**	**#3**	**#4**	**#5**	**Mean**	**S.D.**
0.083	0.7870	0.7620	0.9920	0.8510	0.9090	0.8602	0.0933
0.25	0.5420	0.5750	0.7960	0.7480	0.7500	0.6822	0.1151
0.5	0.5020	0.4890	0.5230	0.4180	0.5760	0.5016	0.0573
1	0.2080	0.3190	0.2330	0.3180	0.3840	0.2924	0.0714
3	0.1640	0.2060	0.3080	0.1660	0.1270	0.1942	0.0695
7	0.0451	0.0699	0.0759	0.0266	0.0440	0.0523	0.0203

Publisher's Note: MDPI stays neutral with regard to jurisdictional claims in published maps and institutional affiliations.

Copyright: © 2021 by the authors. Submitted for possible open access publication under the terms and conditions of the Creative Commons Attribution (CC BY) license (http://creativecommons.org/licenses/by/4.0/).

10	0.0170	0.0330	0.0211	0.0246	0.0081	0.0208	0.0092
24	0.0006	0.0007	0.0005	BQL	0.0005	0.0005	0.0003

BQL, Below the quantification limit, treated as "0".

(C) 5 mg/kg.

	**Plasma concentration (μg/mL)**						
**Time (h)**	**#1**	**#2**	**#3**	**#4**	**#5**	**Mean**	S.D.
0.083	1.1300	3.0400	3.5000	1.8400	2.3775	1.0869	1.1300
0.25	0.9410	2.2800	2.1700	1.1600	1.6378	0.6854	0.9410
0.5	0.6340	1.7400	1.9100	0.9740	1.3145	0.6096	0.6340
1	0.5580	1.3100	1.0800	0.6810	0.9073	0.3489	0.5580
3	0.4870	0.7050	0.7800	0.4270	0.5998	0.1694	0.4870
7	0.3350	0.1360	0.3290	0.1350	0.2338	0.1135	0.3350
10	0.1810	0.0497	0.1990	0.0891	0.1297	0.0718	0.1810
24	0.0074	BQL	0.0140	0.0020	0.0058	0.0063	0.0074

BQL, Below the quantification limit, treated as "0".

Table S2. Plasma concentrations of supinoxin after oral administration of 0.5, 1 and 5 mg/kg in fasted male SD rats.

(A) 0.5 mg/kg.

	**Plasma concentration (μg/mL)**						
**Time (h)**	**#1**	**#2**	**#3**	**#4**	**#5**	**Mean**	S.D.
0.25	0.0382	0.0699	0.0525	0.0484	0.0459	0.0510	0.0118
0.5	0.1280	0.1230	0.0776	0.0843	0.0853	0.0996	0.0239
1	0.0946	0.1610	0.0696	0.0849	0.0880	0.0996	0.0355
3	0.0734	0.0864	0.0452	0.0276	0.0575	0.0580	0.0231
7	0.0185	0.0142	0.0156	0.0027	0.0144	0.0131	0.0061
10	0.0069	0.0047	0.0052	0.0008	0.0076	0.0050	0.0027
24	BQL	BQL	BQL	BQL	BQL	0.0000	0.0000

BQL, Below the quantification limit, treated as "0".

(B) 1 mg/kg.

	**Plasma concentration (μg/mL)**						
**Time (h)**	**#1**	**#2**	**#3**	**#4**	**#5**	**Mean**	S.D.
0.25	0.0846	0.1850	0.2460	0.3710	0.1730	0.2119	0.1060
0.5	0.1680	0.1590	0.2860	0.3870	0.1680	0.2336	0.1006
1	0.1620	0.1660	0.2150	0.3950	0.2180	0.2312	0.0953
3	0.0708	0.1270	0.0944	0.2110	0.1080	0.1222	0.0537
7	0.0042	0.0467	0.0118	0.0526	0.0590	0.0349	0.0250
10	0.0012	0.0170	0.0036	0.0168	0.0298	0.0137	0.0116
24	BQL	BQL	BQL	BQL	0.0016	0.0003	0.0007

BQL, Below the quantification limit, treated as "0".

(C) 5 mg/kg.

	**Plasma concentration (μg/mL)**					
**Time (h)**	**#1**	**#2**	**#3**	**#4**	**Mean**	S.D.
0.25	0.1860	0.5240	0.1150	1.0700	0.4738	0.4357
0.5	0.3310	0.6210	0.5100	1.2400	0.6755	0.3948
1	0.4820	0.6110	0.9490	1.1600	0.8005	0.3102
3	0.3510	0.3300	0.6310	0.6070	0.4798	0.1613
7	0.0722	0.0767	0.1420	0.1600	0.1127	0.0448
10	0.0178	0.0244	0.0587	0.0607	0.0404	0.0225

24	BQL	BQL	0.0008	0.0014	0.0005	0.0007

BQL, Below the quantification limit, treated as "0".

Table S3. Unbound fractions of supinoxin in rat plasma and liver microsomes.

	Unbound fraction (%)[a]	
**Time (h)**	Plasma	Liver microsomes
4	2.30 ± 0.25	51.8 ± 2.5
8	2.83 ± 0.19	57.0 ± 1.7
18	3.63 ± 0.25	62.3 ± 1.6
24	3.54 ± 0.55	61.5 ± 4.6

[a]The data are represented as mean ± SD of triplicate or quadruplicate runs

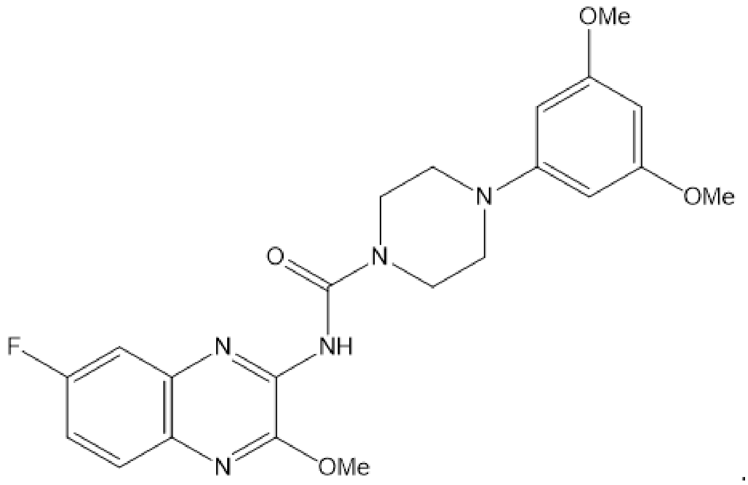

Figure S1. Chemical structure of supinoxin.

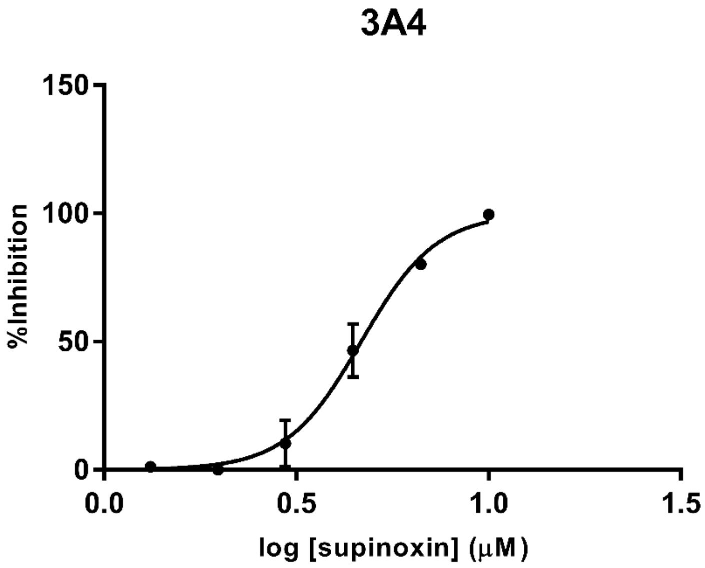

Figure S2. Inhibitory effects of supinoxin on the CYP3A4 activity in human recombinant enzymes. Each point represents the mean ± standard deviation (n = 3).

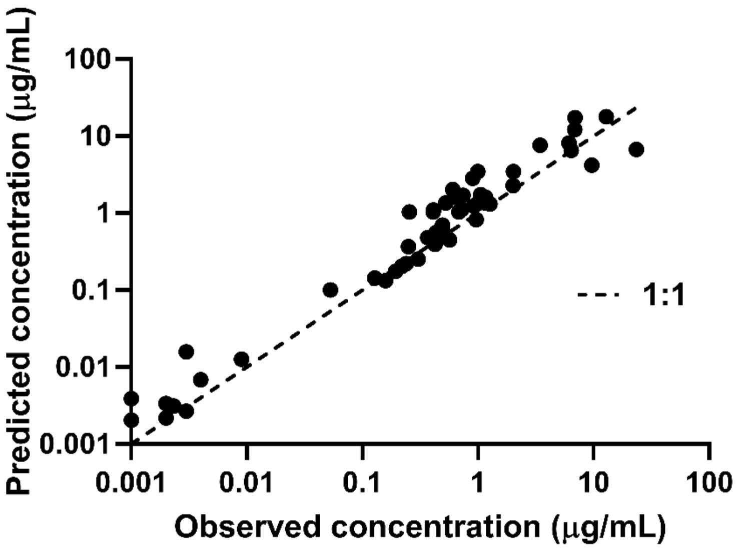

Figure S3. Relationship between observed and predicted concentration.
